# Dynamics understanding of novel solvated drug molecules against emerging *Burkholderia Cepacia* infections in immunocompromised patients

**DOI:** 10.1016/j.rechem.2025.102250

**Published:** 2025-05

**Authors:** Sajjad Ahmad, Faisal Ahmad, Syed Ainul Abideen, Kalsoom Khan, Muhammad Irfan, Farhan Siddique, Norah Abdullah Albekairi, Abdulrahman Mohammed Alshammari, Dong-Qing Wei

**Affiliations:** a Department of Health and Biological Sciences, Abasyn University, Peshawar 25000, Pakistan; b World Health Organization, Park Road, Chak Shahzad, Islamabad 44000, Pakistan; c National Institute of Health. Park road, Chak Shahzad. Islamabad, Islamabad 44000, Pakistan; d Department of Biomedical Engineering, Shanghai Jiao Tong University, Shanghai, China; e Department of Pharmaceutical Chemistry, Faculty of Pharmacy, Bahauddin Zakriya University, Multan 60800, Pakistan; f Department of Pharmacology and Toxicology, College of Pharmacy, King Saud University, Post Box 2455, Riyadh 11451, Saudi Arabia; g ASRT, Inc., Atlanta, Georgia, USA; h Zhongjing Research and Industrialization Institute of Chinese Medicine, Zhongguancun Scientific Park, Nayang, PR China.

**Keywords:** *Burkholderia cepacia*, BBB 25784317, BBB 26580136, Molecular dynamics simulations, Solvation box, Antibacterial activity

## Abstract

The *Burkholderia cepacia* is a Gram-negative bacterium associated with serious infections, especially in immune compromised patients. Recent studies have shown *B. cepacia* to be involved in bloodstream infection in oncology patients. As the bacterium develops resistance to several classes of antibiotics and due to the depleting efficacy of current antibiotics, serious efforts are required to identify new drug targets and unveil novel chemical scaffolds against them. The bacterial tyrosine kinase enzyme takes part in exopolysaccharides production to form a biofilm matrix layer and thus results in the promotion of aggressive and resistant infections. Also, the enzyme has no close homologous copy in the human host, which warrants its targeting by novel anti-virulent compounds. In this work, an in-depth structure based virtual screening process of complete Asinex library, ChemBridge hit2lead database and comprehensive marine natural products database (CMNPD) was performed against tyrosine kinase domain to prioritize promising lead compounds that exhibited the most favorable binding conformation to the domain and achieved stable energy. These three molecules; BBB 25784317, BBB 26580136 and 12,728,806 were highlighted as promising molecules with binding energy scores of −12.58 kcal/mol, −11.52 kcal/mol and − 11.46 kcal/mol, respectively. The control molecule (adenosine diphosphate (ADP)) also showed a strong energy score of −10.88 kcal/mol. To validate the docking results, molecular dynamics simulations in a solvation box were carried out for 100 ns. The investigation reported the systems highly stable in terms of structure secondary structure elements and also in the perspective of intermolecular docked conformation. The mean RMSD of BBB 25784317, BBB 26580136, 12,728,806 and control was 1.30 Å,1.45 Å, 1.53 Å and 1.84 Å, respectively. The intermolecular binding energies predicted by MMPBSA for BBB 25784317, BBB 26580136, 12,728,806 and control were − 57.04 kcal/mol, −49.8 kcal/mol, −47.7 kcal/mol and − 48.63 kcal/mol, respectively. From in silico drug-likeness and pharmacokinetics, the compounds are noticed as high druglike compounds featuring all parameters to be marketed. The compounds were also unveiled as having favorable adsorption, distribution, metabolism, excretion properties, and no toxicity. Experimental antibacterial evaluation of selected compounds showed that compound 12,728,806 revealed antibacterial potency towards Gram-positive and Gram-negative strains. Compound 12,728,806 exhibited zone of inhibition of 8 mm at MIC of 0.4 mg/ml. In a nutshell, the compounds are promising theoretical leads and thus can be subjected to experimental biological activities.

## Highlights

• BBB 25784317, BBB 26580136 and 12,728,806 were highlighted as promising molecules.
• The investigation reported the systems highly stable in terms of intermolecular docked conformation.
• The compounds were noticed as high druglike compounds.

## Graphical abstract

**Figure:**
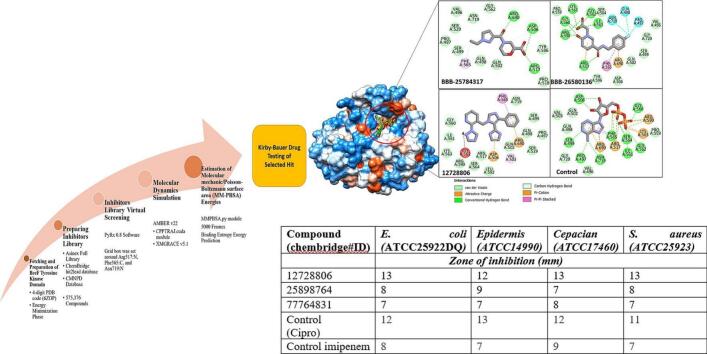

## 1 Introduction

The *Burkholderia cepacia* (in short *B. cepacia)* is a family of opportunistic bacteria with Gram-negative cell wall architecture [1]. The bacteria are predominantly involved in serious infections in patients with compromised immune systems, especially those with cystic fibrosis [2]. Recently, *B. cepacia* has been reported to show severe infections in oncology patients [3,4]. *B. cepacia* often causes bloodstream infections in oncology patients particularly those with central venous catheters (CVCs). The treatment of *B. cepacia* bloodstream infections is difficult to treat due to biofilm production within the catheter. This further complicates the treatment procedure as removing catheters is a hard decision in oncology patients [5]. The enhanced pathogenesis of *B. cepacia* is due to their ability to produce biofilms which safeguard the bacteria from host defenses and disinfectants [6]. The biofilms and the intrinsic antibiotic resistance potential of *B. cepacia* over the last 30 years have resulted in the inefficacy of a large number of antibiotics thus making these bacteria a serious public health concern [7]. To ensure the development of novel antibacterial that target new targets continuous research efforts are needed [8]. In particular, targeting the non-essential biomolecules involved in bacterial virulence holds a promising strategy as it lowers the risk of developing evolutionary pressure on the bacteria. As a result, resistance might not develop [[9], [10], [11]].

The bacterial tyrosine kinases are warranted as promising anti-bacterial targets as they are non-homologous to the human host but also non-essential [2,12]. Moreover, these enzymes are found in both Gram-positive and Gram-negative bacteria respectively and thus can be used as targets of broad-spectrum antibiotic development [6,13]. The tyrosine kinase enzymes modulate various regulatory and metabolic pathways in bacteria such as DNA metabolism, virulence, and polysaccharide biosynthesis [14]. The prime importance of tyrosine kinases as drug targets is their ability to regulate bacterial virulence and biofilm formation [6,15]. There are two types of tyrosine kinases in the prokaryotic systems; odd tyrosine kinases and Hanks-type tyrosine kinases with a characteristic Hanks motif [6,16]. The *bce* gene cluster (*Burkholderia cepacia* exopolysaccharide) in *B. cepacia* is vital for exopolysaccharide biosynthesis [6]. The *bceF* gene (which is part of the bce gene cluster) encodes for the tyrosine kinase domain, which is vital for exopolysaccharide biosynthesis and important to play a role in biofilm formation [6]. In the case of Gram-negative bacteria, the BceF protein is comprised of an N-terminus localized towards the cytoplasm, with an extended transmembrane helix across the membrane, an extracellular domain in the periplasmic space, and a C-terminal catalytic domain [6,17]. The catalytic region of the protein contains Walker A, Walker A' and Walker B motifs [6]. The Walker A motif functions as phosphate binding site while Walker B motif is often found downstream of the Walker A motif and vital for ATP hydrolysis [18,19]. The bacterial tyrosine kinases also have another significant region at the C-terminal which is enriched in tyrosine residues often referred to as Y-cluster [6]. At the bottom line, the bacterial tyrosine kinases differ significantly in the prokaryotic and eukaryotic systems and due to major variations in functional and structural aspects, the bacterial tyrosine kinases are tagged as promising and druggable targets for antibiotics development.

The traditional drug discovery is time consuming and needs extensive resources to ensure the safety and efficacy of novel drug molecules to reach the market [20,21]. A single drug molecule takes upto 14 years and approximately one billion dollars from target identification and regulatory approval [21]. Another aspect to be considered in the pharmaceutical scenario is failure risk [22]. It has been estimated that one or two/10,000 screened molecules can effectively become drugs [21]. To facilitate the rapid discovery of novel drug molecules that require low cost and can be marketed in less time, computer-aided drug design is an area with immense potential [[23], [24], [25]]. The statement can be supported by successful applications of computer-aided drug design such as; oxymorphone which is an agonist of mu-opiniod receptor for treating opioid analgesic, dorzolamide inhibits carbonic anhydrase for managing glaucoma and ocular hypertension [26]. With the potential of computational approaches to rank lead compounds against therapeutic targets, this work sought to perform structure-based drug designing from the Asinex drug library against BceF Tyrosine Kinase Domain and then subject the lead molecules complexes to molecular dynamics simulation studies [27,28]. The rationale behind the selection of complexes is based on molecular docking results. The potent intermolecular binding molecules to the ATP binding sites of the target protein were opted. Furthermore, the selected compounds showed favorable ADMET properties. Thus, considering favorable drug-like properties and pharmacokinetics profile of the shortlisted drug molecules act as strong candidates for in-vitro experiments. Further, the lead molecules were used in binding free energy estimation to re-validate the docking findings. The shortlisted candidates were then used in experimental in vitro investigations.

## 2 Materials and methods

**Fig. 1:**
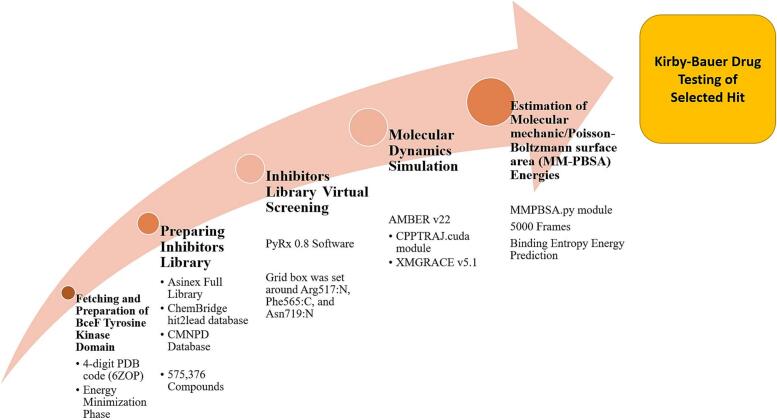
Study flow of the work done in the manuscript.

The flow of study steps is illustrated in Fig.1.

### 2.1 Fetching and preparation of BceF Tyrosine kinase domain

The RCSB protein data bank, also known as PDB, harbors the 3D structure of proteins and other macro and micro-biomolecules [29]. The BceF Tyrosine Kinase Domain crystal structure was fetched from the PDB databank in Chimera v 1.17 by putting the 4-digit code of 6Z0P in the fetch structure option of the software [6,30]. The enzyme sequence was taken from *B. cepacia* and expressed in the *Escherichia coli* BL21 (DE3) strain. No mutation was done during the expression and crystallization process. The crystal structure was released on 14 April 2021 with the following experimental data snapshot; the crystal structure was determined by X-RAY diffraction method, resolved at 1.85 Å with R-value observed of 0.183, R-value work of 0.182 and R-value free of 0.202. After fetching the structure, the missing hydrogen atoms were added using AddH tool in Chimera v 1.17, and Gasteiger charges were assigned [30]. The structure was then utilized in energy minimization phase to refine structural errors. The steepest descent and conjugate gradient algorithms (steps = 3000) were applied to free the structure from steric clashes [31]. The AutoDock tools 1.5.7 was then considered to convert the enzyme structure into ‘pdbqt’ format [32].

### 2.2 Preparing inhibitors library

The Asinex full library (https://www.asinex.com/screening-libraries-(all-libraries)), ChemBridge hit2lead database (https://www.hit2lead.com/) and comprehensive marine natural products database (CMNPD) were employed for virtual screening process against the targeted BceF Tyrosine Kinase Domain. The library has been created by utilizing DNA encoded chemistry with knowledge of macrocycles, computational small molecules design and cellular permeability. The mentioned points ensure the creation of a viable database with diverse chemical scaffolds from different natural and chemical sources. The full Asinex library contains about 575,376 compounds. The library can be further split into BioDesign library (that incorporates chemical structures with known pharmacological biopotency), Elite and Synergy library (with favorable ADMET properties), and Gold and Platinum library (have high drug-likeness and in accordance to Lipinski rule of five [33]). The Asinex library was imported in .sdf format to the PyRx 0.8 software, where the molecules were extensively energy minimized by MM2 force field [34,35]. Afterward, the molecules were converted into .pdbqt format. The (adenosine diphosphate (ADP)) was used as a control to compare the binding affinity of the lead molecules.

### 2.3 Inhibitors library virtual screening

To virtually screened the prepared inhibitors library against BceF Tyrosine Kinase Domain with aim to identify promising leads showing robust stability and least binding energy score, structure based virtual screening was conducted. The screening was performed using PyRx 0.8 software, where AutoDock Vina 4.1 was employed [34,36]. During the screening process, the grid box was set around Arg517:N, Phe565:C, and Asn719:N [6]. The grid box dimensions along XYZ coordinates were fixed at 30 Å. Each molecule conformations generated with the receptor was 100. We prioritized the complexes with the best docked molecules, which exhibited the minimum binding energies affinity, for further downstream analyses. The docking results were validated by GOLD docking software v 5.1 by employing the same docking parameters discussed above for the AutoDock Vina 4.1 [37]. The compounds were ranked by GOLD fitness score. The higher the docking fitness score, higher the binding affinity of the compound is.

### 2.4 Molecular dynamics simulation

**Fig. 2:**
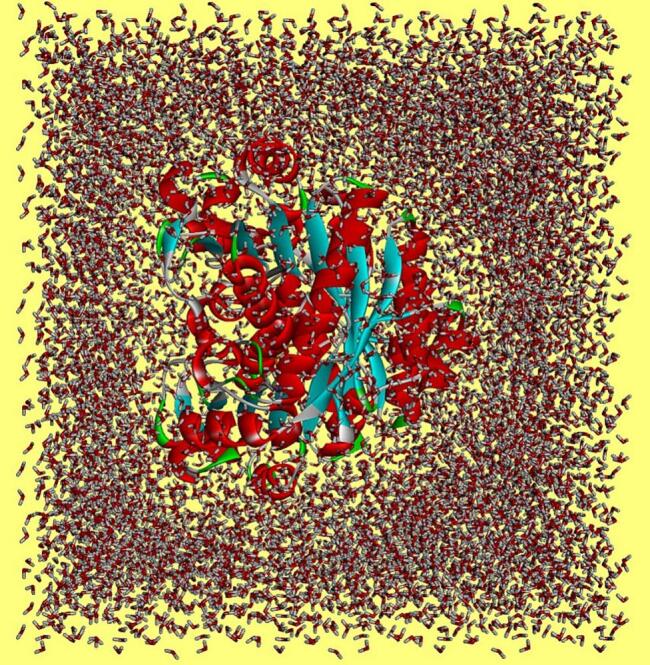
Representative docked compound complex with the BceF tyrosine kinase domain in water box.

To track structure dynamics and intermolecular docked binding mode stability of the selected docked complexes, molecular dynamics simulations were carried out. The AMBER v22 simulation program was used to complete the simulation protocol where the topology files were generated first via Antechamber program [38,39]. The FF19SB was employed as a force field to estimate the forces between the atoms of the molecules and also between the molecules [40]. This force field was considered to parameterize for BceF Tyrosine Kinase Domain. On the other hand, the GAFF2 was taken into consideration for the inhibitor ligands [41]. The selected docked solutions were submerged into the OPC water model (Fig.2); where counter ions enough to neutralize the systems were added [42]. The energy minimization of the docked submerged complexes was achieved to discard any undesirable contact and steric conflicts. The steepest descent and conjugate gradient algorithms were used for this purpose with number cycles set to 50,000. Next, the NVT ensemble was considered to keep the number of atoms, volume and temperature of the complexes constant. Further, the pressure of complexes was maintained using NPT ensemble. The temperature was set to 300 K and pressure was set to 1 bar. The systems underwent a 1 ns equilibration period, followed by a 100 ns production run. During this time, the temperature was controlled using the Langevin dynamics algorithm, while hydrogen bonds were maintained at a constant state using the SHAKE algorithm [43,44]. The Particle Mesh Ewald (PME) was used to handle long term electrostatic interaction [45]. The CPPTRAJ.cuda module was applied on the simulation trajectories to calculate the structure deviation over the simulation time [46]. The statistical plotting based on the simulation trajectories was done using XMGRACE v5.1 [47].

### 2.5 Estimation of molecular mechanic via binding free energies

The MM-PBSA binding free energies estimation is often used in conjugation with the molecular simulation results to better conclude stability of docked complexes [48,49]. The MM-PBSA estimates binding free energies for complex, receptor and ligand using the following equation.

∆G binding=Gcomplex–Greceptor–Gligand

The MM-PBSA calculations were run on the simulation trajectories using MMPBSA.py module [50]. During the calculation, 5000 frames were collected along the simulation length with equal time intervals.

### 2.6 Binding entropy energy prediction

The contribution of binding entropy energy to the total MM-PBSA was determined using AMBER normal mode entropy estimation method [51]. This method is computationally very expensive and thus only small set of frames (5 in number) were analyzed only. The entropy energy is calculated by three different energy terms; rotational, vibrational and translational. All the terms are estimated in kcal/mol.

### 2.7 Absorption, distribution, metabolism, excretion and toxicity (ADMET) analysis

The ADMET properties of selected lead molecules were investigated using SwissADME online server [52]. The SwissADME is a free and comprehensive tool for evaluating compounds physicochemical, water solubility, lipophilicity, pharmacokinetics, druglikeness, and medicinal chemistry properties. The toxicity of compounds was determined using pKCSM online server [53].

### 2.8 Antibacterial assay

Three compounds (Chembridge#ID 12728806, 25898764, and 77764831) from Table 1 (provided by Dr. Faisal, NIH, Islamabad, Pakistan) were assessed for antibacterial efficacy using the agar disc diffusion technique [54]. The Mueller Hington Agar (MHA) medium was used as the culturing media for testing purposes [55]. The compound stock solutions were created by dissolving 1 mg of each inhibitor in 1 ml of DMSO (dimethyl sulfoxide, Duksan pure chemicals, Korea). Ciprofloxacin served as the positive control, and its stock solution was made by dissolving 1 mg of ciprofloxacin powder in 1 ml of DMSO. The bacterial test strains inoculum was generated by introducing new test strains inoculum into sterilized 1 ml of normal saline solution. The turbidity of the inoculum was then calibrated to match the 0.5 McFarland standard using a solution containing 1 % BaCl2 and 1 % H2SO4. Four ATCC bacterial strains were used in the experiment: two Gram-positive strains (*Staphylococcus aureus* (ATCC25923) and *Staphylococcus epidermis* (ATCC14990), two Gram-negative strains (*Escherichia coli* (ATCC25922DQ) and *B. cepacia* (ATCC17460). A uniform layer of bacteria was created by using sterilized cotton swabs. Sterilized filter paper discs (6 mm in diameter, Whatman International Ltd., England) were positioned on the plates at a 90° angle. The compound samples, positive control (5 μl, final concentration 5 μg/5 μl or 1 μg/μl), and negative control were administered onto the discs. The plates were placed in an incubator for 24 h (temperature, 37 °C). The antibacterial activity potency of tested compounds was assessed by measuring the zone of inhibition in millimeters (mm). The antimicrobial test was conducted in triplicate, and three values were statistically analyzed to calculate the means and standard deviation (SD).

**Table 1:** Compounds ranked from different databases reported robust binding energy for the receptor in kcal/mol.

Rank	Compounds	Database	Chemical Structure	Binding Energy Score in Kcal/mol	GOLD Score
1	BBB-25784317	Asinex		−12.58	98.20
2	BBB 26580136	Asinex		−11.52	92.17
3	12,728,806	ChemBridge hit2lead		−11.46	90.59
4	25,898,764	ChemBridge hit2lead		−11.01	88.69
5	CMNPD20	CMNPD		−9.14	85.01
6	CMNPD1540	CMNPD		−9.06	84.11
7	CMNPD31329	CMNPD		−8.51	81.12
8	CMNPD1513	CMNPD		−8.01	76.92
9	7,277,764,831	ChemBridge hit2lead		−7.55	72.78
10	CMNPD4175	CMNPD		−6.51	68.83

## 3 Results and discussion (computational analysis and experimental validation) computational analysis

### 3.1 Docking analysis

**Fig. 3:**
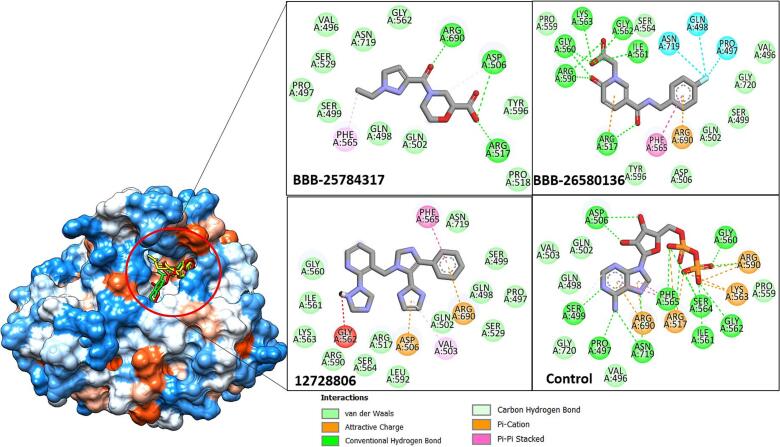
The virtual screened docked leads in the active pocket of tyrosine kinase domain. The control, BBB-25784317, BBB-26580136 and 12,728,806 are presented in red, yellow, red sticks and orange, respectively. The chemical interactions of the compounds to the enzyme active site residues are also shown. (For interpretation of the references to colour in this figure legend, the reader is referred to the web version of this article.)

Structure-based virtual screening of drug-like compounds from the Asinex library was done against the tyrosine kinase domain to predict compounds that revealed the best binding mode to the target enzyme. The Lipinski rule of five identified 50,784 as drug-like while the remaining as non-druglike. The screening process noticed two compounds; BBB-25784317, BBB-26580136, and 12728806 as the best binders of the enzyme **(**Table 1**)**. The selection of these three compounds was based on the fact that both compounds on repeated runs illustrated stronger binding than others. BBB 25784317, BBB 26580136 and 12728806 binding energy scores was −12.58 kcal/mol, −11.52 kcal/mol, and − 11.46 kcal/mol, respectively. The control molecule also showed a strong energy score of −10.88 kcal/mol. The binding energy score of the leads and control are figured in S-Fig.1.Chemically, BBB 25784317, BBB-26580136, 12728806 and control is 4-(1-ethyl-1H-pyrazole-3‑carbonyl)morpholine-2-carboxylic acid, 1-(carboxymethyl)-5-((4-fluorobenzyl)carbamoyl)pyridin-1-ium-2-olate, 3′-((2-(1H-1,2,4-triazol-1-yl)pyridin-3-yl)methyl)-5′-phenyl-1H,3′H-2,4′-biimidazole and (5-(6-amino-9H-purin-9-yl)-3,4-dihydroxytetrahydrofuran-2-yl)methyl trihydrogen diphosphate, respectively. Both the leads and control molecule showed binding to same site which Arg517, Phe565, and Asn719 constitute. The compounds reported binding to the pocket bottom showing robust chemical interactions with key active site residues such as Arg690, Aps506, Arg517, Lys563, Gly560, Arg590, Ile561, Ser564, Gly562, Asn719, Pro497, and Ser499 **(**Fig.3**)**. In case of BBB 25784317, 4-formylmorpholine-2-carboxylic acid formed to play major contribution in term of hydrogen bonds with residues Asp506 and Arg690 while the opposite 1-ethyl-1H-pyrazole ring is engaged in hydrophobic contacts with Asn719, Val496, Ser529, Pro497, Ser499 and Tyr596. The prominent chemical moiety of BBB 26580136 was 1-(carboxymethyl)-5-(methylcarbamoyl)pyridin-1-ium-2-olate which played a vital role in stabilizing the compound to the active pocket. This chemical region formed strong short distance hydrogen bonds with Arg517, Gly560, Gly562, Lys563, and Arg590. The terminal fluorobenzene ring produced interaction with Pro497, Gln498, and Asn719. On the other side, the control molecule along the length of the structure generates strong hydrophilic and hydrophobic bonds. The docking results presented here indicated promising binding affinities of the compounds with the target protein. The cross-validation of the AutoDock Vina 4.1 results by the GOLD fitness score is also presented in Table 1. Both the docking results complement each other in ranking the compound's affinity for the receptor protein.

### 3.2 Intermolecular conformation structure dynamics and residues flexibility

The docking findings are often validated by molecular dynamics simulation as the former give static intermolecular docked conformation while the latter is based on dynamics simulation trajectories. The structure deviations or stability of BceF Tyrosine Kinase Domain in the presence of lead molecules/control molecule was determined using the following statistical parameters; root mean square deviation (RMSD), root mean square fluctuation (RMSF) and radius of gyration (Rg). All these analyses were done in angstroms (Å) and are based on carbon alpha atoms.

#### 3.2.1 RMSD analysis

**Fig. 4:**
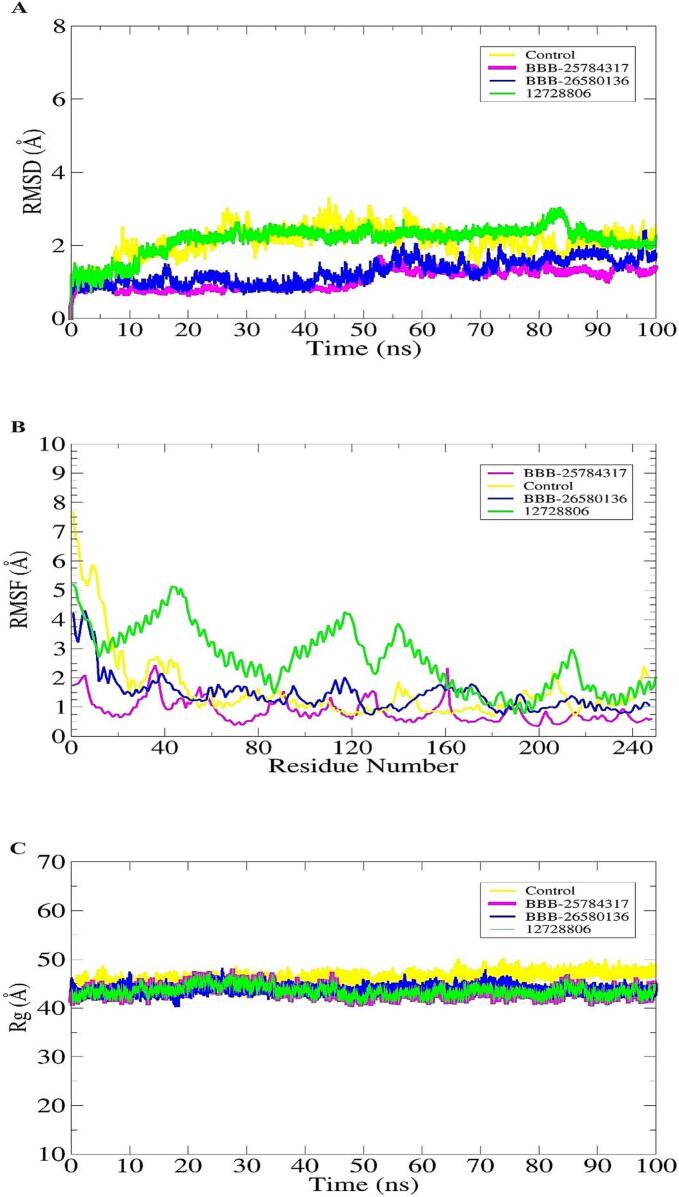
Physical structure dynamics of docked complexes. **A.** RMSD, **B,** RMSF and **C.** Rg**.** All the structure analysis was done in angstroms.

The RMSD allows superimposition of simulation frames over the initial docked structure which was used as a reference [56, 57]. The RMSD is a significant indicator to predict receptor structure dynamics over the simulation time. It further can guide us about the docked intermolecular conformation of the complexes. Higher RMSD and lower RMSD illustrates more structure variation and low structure variations, respectively. The mean RMSD of BBB 25784317, BBB 26580136, 12,728,806 and control was 1.30 Å,1.45 Å, 1.53 Å and 1.84 Å, respectively **(**Fig.4A**)**. By this analysis, all the complexes in particular the lead complexes were seen in high structure stability than the control system. Though neither any major global nor local changes were noticed, but towards the simulation end, all the three systems were seen well converged with stable RMSD plot.

#### 3.2.2 RMSF analysis

The RMSF tells about the receptor molecule residues level fluctuations and stable nature during the course of simulation time [58]. It also highlights the residues involved in stable binding of the ligand molecules. The mean RMSF value of the BBB 25784317, BBB 26580136, 12,728,806 and control was 1.34 Å, 2.34 Å, 2.94 Å and 3.42 Å, respectively **(**Fig.4B**)**. The N-terminal of the receptor molecule showed higher variations compared to the central and C-terminal regions. Most of the residues that were seen involved in ligands interactions were found highly stable demonstrating their significant role in intermolecular docked conformation stability. The localized fluctuations were seen due to high loop percentage in the protein, which were observed not to affect overall ligand binding mode and interactions network.

#### 3.2.3 Rg analysis

Next, to decipher docked complexes compact/relax nature in the simulation time frame, Rg analysis was performed [59]. Higher compact structure indicates formation strong and stable complexes. The mean Rg value of BBB 25784317, BBB 26580136, 12,728,806 and control was 32.5 Å, 33.67 Å, 32.88 Å and 35.01 Å, respectively **(**Fig.4C**)**. The values illustrate the systems are highly compact due to strong intermolecular interactions between the protein and docked compounds. This further support the fact that the compounds showed stable binding conformation with the enzyme and could be potential lead molecules to block the enzyme function. These results are also aligned with the RMSF that revealed stable dynamics of the protein in the presence of compounds.

#### 3.2.4 Solvent accessible surface area (SASA) analysis

**Fig. 5:**
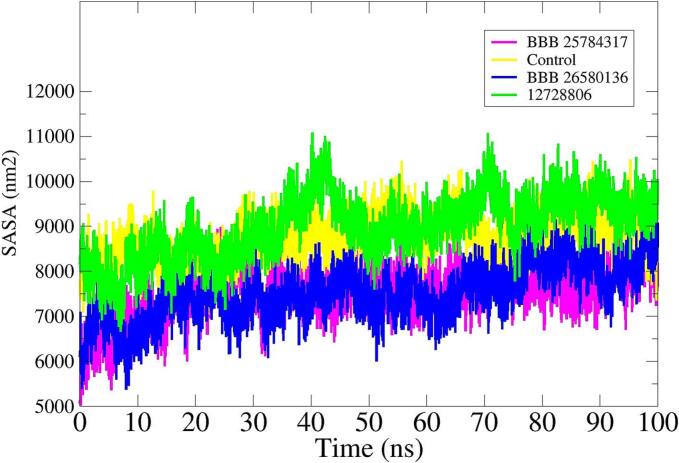
SASA analysis for the simulated systems.

SASA analysis demonstrates the area of protein exposed to interactions with neighboring solvent molecules. All three systems were noticed to have a larger area exposed for interactions with the water molecules present in the surroundings. This also highlights the role of water molecules in interactions with the protein active site residues and compounds. The mean SASA value of control, BBB-25784317, BDB-26580136, and 12,728,806 were 8300 nm^2^, 6900 nm^2^, 7400 nm^2^ and 8214 nm^2^, respectively (Fig.5).

### 3.3 Estimation of MM-PBSA energies

The validation of docking results is vital as docking predictions are often false positive and false negative, therefore, a more reliable method is needed to investigate the intermolecular binding energies of docked complexes [60]. The docking often gives static snapshot of ligand binding mode to the receptor. On the other hand, the MM-PBSA/MM-GBSA binding free energies estimation are done on simulation trajectories where hundreds and thousands of frames are used and docked energies are estimated [61,62]. The MM-PBSA employs the Poisson-Boltzman equation to calculate the free energy whereas the MM-GBSA considers generalized born approximation, which is a faster treatment of the Poisson-Boltzman Eq. [49]. Likewise, the van der Waals energy of the complexes was noticed as the most dominating where the BceF Tyrosine Kinase Domain residues played considerable contribution through hydrophobic contacts. The net van der Waals energy of BBB 25784317, BBB 26580136, 12,728,806 and control reported was −52.36 kcal/mol, −50.08 kcal/mol, −48.12 kcal/mol and − 49.63 kcal/mol, respectively. Similarly, the columbic contribution to overall docked complexes were also significant. The net electrostatic energy of BBB 25784317, BBB 26580136, 12,728,806 and control was −20.21 kcal/mol, −16.94 kcal/mol, −18.01 kcal/mol and − 12.64 kcal/mol, respectively. Both the van der Waals and electrostatic energies are promising in term of making the complexes stable both in aspect of interaction energies and binding conformation. The solvation energy of MMGBSA in all complexes was found more compared in the MMPBSA. Also, the MMGBSA net binding energies.

were noticed low than those reported by MMPBSA. The net MMGBSA binding energy of BBB 25784317, BBB 26580136, 12,728,806 and control was - 56.24 kcal/mol, −48.93 kcal/mol, −47.02 kcal/mol and - 46.65 kcal/mol, respectively. Similarly, the net MMPBSA energy generated for the complexes were; BBB 25784317 (−57.04 kcal/mol), BBB 26580136 (−49.8 kcal/mol), 12,728,806 (−47.7 kcal/mol) and control (−48.63 kcal/mol). All the mentioned values strongly indicate the formation of stable complexes and both hydrophilic and hydrophobic energies played important roles compounds binding to the receptor. More details of the binding free energies of docked complexes can be found in Table 2.

**Table 2:** The MM-PBSA energies of complexes estimated in kcal/mol.

Energy Parameter	BBB 25784317	BBB 26580136	12,728,806	Control
MMGBSA				
Van der Waals	−52.36	−50.08	−48.12	−49.63
Columbic	−20.21	−16.94	−18.01	−12.64
Gas phase	- 72.57	- 67.02	−66.13	- 62.27
Solvation	16.33	18.09	19.11	15.62
Net	- 56.24	−48.93	−47.02	- 46.65
MMPBSA				
Van der Waals	−52.36	−50.08	−48.12	−49.63
Columbic	−20.21	−16.94	−18.01	−12.64
Gas phase	- 72.57	- 67.02	−66.13	- 62.27
Solvation	15.53	17.22	18.43	13.64
Net	−57.04	−49.8	−47.7	−48.63

### 3.4 AMBER normal mode entropy investigation

The entropy energy contribution in net MMPBSA and MMGBSA binding energy was not calculated in the above step [63]. This was due to high computational power needed to run both the MMPBSA and entropy calculations at same time. The net entropy energy contribution of the systems was estimated separately using AMBER normal mode entropy analysis. The lower entropy energy is preferred as high entropy energy illustrates more intermolecular freedom and thus the systems receptor and ligand are likely be dissociated from one another. The entropy energy of the complexes is plotted in S-Fig.2. As can be seen in the figure, BBB 25784317, BBB 26580136, 12,728,806 and control binding entropy energy is 5.36 kcal/mol, 6.98 kcal/mol, 9.2 kcal/mol and 15.08 kcal/mol, respectively. The control system was seen to have more freedom energy compared to the lead systems.

### 3.5 WaterSwap calculations

We performed a WaterSwap study to validate the MMPBSA binding free energies [64]. To further confirm the complexes' intermolecular binding free energies, the WaterSwap was carried out. The WaterSwap swaps water cluster present in the BceF Tyrosine Kinase Domain active pocket with the docked ligand. Three algorithms were used; free energy perturbation (FEP), thermodynamic integration (TI) and Bennett's [65,66]. The predicted score of all three algorithms are given in S-Fig.3**.** The BBB 25784317 was found as the most stable system with FEP, TI and Bennett's score of −47.05 kcal/mol, −46.35 kcal/mol, and − 47.1 kcal/mol, respectively. The lead molecules were reported more stable than the control system.

### 3.6 Drug-likeness analysis

Drug-likeness are set of properties shared by the compounds and existing drugs. The drug-like molecules have high percentage to be converted into successful drugs. In this analysis, five drug rules were used; Veber [67], Ghose [68], Muegge [69], Egan [70] and Lipinski rule of five [33] **(**Table 3**)**. The BBB-25784317 molecule showed fulfillment of most the drug-like rules except the Ghose rule. The BBB-26580136 on the other hand illustrated to fulfill all the drug-like rules. The 12,728,806 was only reported to violate the Muegge rule while passing all others. In contrast, the control molecule was found not to follow all the presented drug-like rules. All the given drug-like rules are vital in predicting the good properties a given compound must possess to be branded. The bioavailability score of the compounds was next determined. The bioavailability score determines 10 % oral bioavailability based on potential surface area and Lipinski rule of five. All the lead molecules showed a good bioavailability score compared to the control. The bioavailability score of BBB-25784317, BBB-26580136, and 12,728,806 is 0.56, 0.56, and 0.55, respectively.

**Table 3:** Drug-like rules and bioavailability score evaluated for the compounds.

Druglikeness	BBB-25784317	BBB-26580136	12,728,806	Control
Veber	Yes	Yes	Yes	No
Ghose	No; 1 violation: WLOGP<-0.4	Yes	Yes	No
Muegge	Yes	Yes	No	No
Egan	Yes	Yes	Yes	No
Lipinski	Yes; 0 violation	Yes; 0 violation	Yes; 0 violation	No
Bioavailability Score	0.56	0.56	0.55	0.11

### 3.7 Prediction of pharmacokinetic properties

The pharmacokinetic properties of leads and control were evaluated to predict the compounds ADME behavior [71]. The details of pharmacokinetic prediction are tabulated in Table 4. Both the leads were found to show high gastrointestinal absorption (GI) compared to the control that showed low score for the GI [72]. The leads and control were further noticed non-permeable through the blood brain barrier (BBB) [73]. This ensures that the compounds do not cause any unwanted effects and have no adverse effects on the central nervous system. The compounds and control also were classified as non-substrate for the p-glycoprotein (p-gp) which illustrates the compounds can't be expelled from the cells and thus drug concentration will be available for therapeutic impact [74]. Interestingly, the leads and control are non-inhibitor of cytochrome enzymes family thus easing the compounds metabolism. The skin permeability of BBB-25784317, BBB-25784317, 12,728,806 and control was found as −8.14 cm/s, −7.07 cm/s, −6.34 cm/s and − 12.19 cm/s, respectively. The skin permeability of the lead structures was better than the control.

**Table 4:** Computational pharmacokinetic studies of selected compounds and control.

Pharmacokinetics	BBB-25784317	BBB-26580136	12,728,806	Control
GI absorption	High	High	High	Low
BBB permeant	No	No	No	No
P-gp substrate	No	No	No	No
CYP1A2 inhibitor	No	No	No	No
CYP2C19 inhibitor	No	No	No	No
CYP2D6 inhibitor	No	No	No	No
CYP3A4 inhibitor	No	No	No	No
Log *K*_p_ (skin permeation)	−8.14 cm/s	−7.07 cm/s	−6.34 cm/s	−12.19 cm/s

### 3.8 Water solubility analysis

Water solubility analysis is a key phase in drug development [65]. Good water solubility makes sure that the given compound reaches in high concentration to the target site and perform therapeutic effects efficiently. Water solubility predictions were done using three methods such as Ali method [75], SILICOS-IT method [75], and ESOL method [75]. All three methods suggested both the leads and control as highly soluble to soluble. The Ali solubility method uses the topographical polar surface area (TPSA) as a replacement of the melting point descriptor of the general solubility equation. This allows improved aqueous solubility prediction by 6.6 % [76]. The SILICOS-IT approach linear correlation coefficient is corrected by molecular weight which is R^2^ = 0.75 [75]. The ESOL (Estimated Solubility) model predicts compound solubility from its structure. This method calculates linear regression against nine molecular properties to measure a given compound's solubility. The properties include LogPoctanol, molecular weight, number of rotatable bonds, and heavy atoms in aromatics systems [77].

### 3.9 Medicinal chemistry and toxicity analysis

Medicinal chemistry analysis was further conducted to shed light on whether the compounds are suitable from medicinal perspective in light of the following parameters such as pan-assay interference compounds (PAINS), brenk, lead-likeness [78], and synthetic accessibility. As a result, the compounds can only attach to a single biomolecule, decreasing the likelihood that they would bind to various targets. The lead compounds and control exhibited no alteration for PAINS [79]. Similarly, BBB-25784317 only showed zero alert for brenk [75]. This illustrates the compound to be metabolically stable, with an accepted level of toxicity and is metabolically stable. Furthermore, the leads were disclosed to follow lead-likeness ability while the control was disobeying this rule. The synthetic accessibility score predicts compound synthesis ease and is correlated to chemical fragment frequency. The synthetic accessibility score ranges from 1 to 10 for very easy to synthesize and hard to synthesize, respectively [75,80]. The BBB-25784317, BBB-25784317, 12,728,806, and control synthetic accessibility score was 2.78, 2.26,3.20 and 4.74, respectively. The values predicted the lead molecules to be more feasible for chemical synthesis than the control. Further, the selected lead compounds were reported to show no AMES toxicity, immunogenicity, hepatotoxicity, and mutagenicity.

## 4 Experimental validation

### 4.1 Antibacterial biological potency evaluation of selected compounds

Characterization of antibiotic resistance at the primary level with control antibiotics and commercially available inhibitors (ciprofloxacin and imipenem) were evaluated against gram-positive as well as gram-negative isolates from the ATCC. As per the criteria set by the Clinical and Laboratory Standards Institute (CLSI), bacterial susceptibility is classified into three categories: susceptible, intermediate, and resistant. These categories are determined based on the zone of inhibition (ZOI) measurements, with ZOI values of ≥20, =15–19, and ≤ 14, respectively (Khan et al., 2021). The results indicated that the bacterial isolates exhibited high susceptibility to antibiotics. Except for a small number, the majority of bacterial isolates shown either intermediate susceptibility or resistance to ciprofloxacin and imipenem. Table 5 and Table 6 provide more details. The zone of inhibition (ZOI) for Gram-positive bacteria ranged from 0 to 13 mm, while for Gram-negative bacteria it ranged from 0 to 13 mm for compound 12,728,806 followed by compound 25,898,764 and compound 77,764,831. These results were then further assessed by a minimum inhibitory concentration of the compound 12,728,806 against gram-positive as well as gram-negative bacterial cultures **(**Table 6**)**. Results showed that the compound 12,728,806 that was chosen during computational screening showed antibacterial potency against gram-positive as well as gram-negative isolates. Compound 12,728,806 exhibited antibacterial activity against *E. coli (*ATCC25922DQ*)*, *S. epidermis (ATCC14990, B. cepacia (ATCC17460 and S. aureus (ATCC25923)* of 8 mm at MIC of 0.4 mg/ml **(**Table 6**)**. The observed antibacterial effects in planktonic cultures were compared with molecular docking and molecular dynamics simulation data. The comparative analysis revealed homology between computational and experimental findings in term of antibacterial activity of the compound. The binding affinity of shortlisted compounds and possible inhibitory interactions with the BceF kinase domain are somewhat to be explored in-depth through isothermal titration calorimetry, surface plasmon resonance, biolayer interferometry, or fluorescence polarization techniques. Additional studies in biofilm-forming conditions together with specific assays including phosphotransferase activity measurements, exopolysaccharide quantification and enzyme inhibition assays could improve the direct link to BceF inhibition.

**Table 5:** Antibacterial activity of selected compounds against ATCC bacterial strains.

Compound (chembridge#ID)	*E. coli (*ATCC25922DQ*)*	*Epidermis (ATCC14990)*	*Cepacian (ATCC17460)*	*S. aureus (ATCC25923)*
	*Zone of inhibition (mm)*			
12,728,806	13	12	13	13
25,898,764	8	9	7	8
77,764,831	7	7	8	7
Control (Cipro)	12	13	12	11
Control imipenem	8	7	9	7

**Table 6:** MIC concentration of compound 12,728,806 was used against ATTC bacterial strains.

S. No.	MIC concentration	*E. coli*	*S. epidermis (ATCC14990*	*B. cepecia*	*S. aureus (ATCC25923)*
12,728,806	1 mg/ml	12	14	12	10
12,728,806	0.8 mg/ml	11	13	11	9
12,728,806	0.6 mg/ml	10	11	9	8
12,728,806	0.4 mg/ml	8	9	8	7
12,728,806	0.2 mg/ml	0	0	0	0
12,728,806	0.1 mg/ml	0	0	0	0
Control (Cipro)	5 μg/ml	11	13	12	11
Control imipenem	10 μg/ml	8	7	9	7

### 4.2 Synergistic assay

The synergistic experiment assessed the antibacterial effectiveness of computationally selected compound 12,728,806 when combined with ciprofloxacin, imipenem and amoxicillin + clavulanic against ATCC bacterial isolates. The combination study revealed that the compound 12,728,806 + ciprofloxacin exhibited enhanced activity, with a mean zone of inhibition of 21 mm against *E. coli,* 19 mm against *S. epidermis, 20* mm against *B. cepecia* and 21 mm against *S. aureus*. Nevertheless, a significant advancement in combating this compound demonstrated promising results. The study also assessed the combined impact of the minimum inhibitory concentrations (MIC) of compound 12,728,806 with ciprofloxacin, imipenem, and amoxicillin-clavulanic acid against bacterial isolates. The results are shown in Table 7. The results indicated that all four bacterial strains exhibited a maximal zone of inhibition at the minimal inhibitory dose. The typical mean zone of inhibition was about 22 mm against *E. coli*, approximately 20 mm against *S. epidermis*, 21 mm against *B. cepecia* and approximately 22 mm in response to *S. aureus*. The lowest inhibitory level was 5μg + 1μg (ciprofloxacin + compound 12,728,806). This reaction seems to be more efficacious when compared to the positive control of amoxicillin + clavulanic acid. However, when imipenem and compound 12,728,806 were combined at a concentration of 10μg + 1μg of MIC, they exhibited a significant reaction, as shown by an average mean inhibition zone of around ±24 mm. The selected 12,728,806 showed a synergistic effect when used in combination with Amoxicillin and Ciprofloxacin. The exact molecular mechanism of this is not documented in this study due limited scope of the research done herein. However, the possible mechanisms may include: (1) BceF inhibition interfering with exopolysaccharide synthesis enhancing permeability of bacterial membranes, (2) disrupting bacterial stress responses making cells more sensitive to antibiotics; as well as (3) simultaneously targeting BceF and other similar tyrosine kinases that are vital for bacterial defenses.

**Table 7:** Synergistic effect of compound 12,728,806 together with ciprofloxacin and imipenem antibiotics.

Bacterial strains	Ciprofloxacin + 12,728,806 (5μg + 1μg)	Imipenem + 12,728,806 (10μg + 1μg)	Amoxicillin + Clavulanic Acid (+ve Control) (20 + 10) μg/ml	DMSO (−ve Control)
Zone of inhibition (mm)				
***E. coli***	±21	±22	±24	0
***S. epidermis***	±19	±20	±22	0
***B. cepecia***	±20	±21	±20	0
***S. aureus***	±21	±22	±24	0

## 5 Concluding remarks

In this study, three compounds named BBB 25784317, BBB 26580136 and 12,728,806 were unveiled as promising inhibitors of the bacterial tyrosine kinase domain in *B. cepacia* catheter–related infections in cancer patients. The compounds chemical structure is different from the existing antibiotics and might enriched novel drugs arsenal against the bacterial pathogens. The compounds very proved by several computational approaches to show stable docked intermolecular conformation and interaction energies. Experimental validation is done to unveil biological potency of the compound 12,728,806. The BceF is predominantly present in *B. cepacia*. As presented, the top hits offered broad-spectrum antibacterial activity. This can be explained by the presence of similar protein tyrosine kinases/having somewhat similar binding pockets in bacteria which are not only highly conserved across a wide range of bacterial species but also crucial for regulating polysaccharide biosynthesis and export. These events are vital for bacterial biofilm and capsule formation, as well as bacterial virulence [14]. Further experimental validation of all listed compounds is necessary to decipher inhibition mechanism behind actual biological potency of the selected compounds. Enzyme inhibition assays are highly suggested to investigate the compounds binding affinity with the enzyme. Additionally, invitro and animal models' studies can be highly useful to test the compound's anti- tyrosine kinase activity.

## CRediT authorship contribution statement

**Sajjad Ahmad:** Writing – review & editing, Writing – original draft, Visualization, Validation, Supervision, Software, Resources, Project administration, Methodology, Investigation, Funding acquisition, Formal analysis, Data curation, Conceptualization. **Faisal Ahmad:** Writing – review & editing, Methodology, Investigation, Data curation. **Syed Ainul Abideen:** Writing – review & editing, Writing – original draft, Methodology, Investigation, Formal analysis, Data curation. **Kalsoom Khan:** Writing – review & editing, Writing – original draft, Methodology, Investigation, Formal analysis, Data curation. **Muhammad Irfan:** Writing – review & editing, Writing – original draft, Methodology, Investigation, Data curation. **Farhan Siddique:** Writing – review & editing, Writing – original draft, Methodology, Investigation, Data curation. **Norah Abdullah Albekairi:** Writing – review & editing, Methodology, Investigation, Formal analysis, Data curation. **Abdulrahman Mohammed Alshammari:** Writing – review & editing, Resources, Methodology, Investigation, Formal analysis, Data curation. **Dong-Qing Wei:** Writing – review & editing, Writing – original draft, Validation, Methodology, Investigation, Formal analysis, Data curation.

## Funding

Authors are thankful to the Researchers Supporting Project number (RSPD2025R1035), King Saud University, Riyadh, Saudi Arabia.

## Declaration of competing interest

The authors declare that they have no known competing financial interests or personal relationships that could have appeared to influence the work reported in this paper.

## Data Availability

Data will be made available on request.

## Appendix A Supplementary data

Supplementary material Image 11
