# Supplementary material for: Dynamics understanding of novel solvated drug molecules against emerging Burkholderia Cepacia infections in immunocompromised patients
Source: Results Chem. 2025 May;15:None. doi: 10.1016/j.rechem.2025.102250 (PMC12149026; doi:10.1016/j.rechem.2025.102250)
Supplement: Supplementary file 1 — Supplementary material [file mmc1.docx]

**Dynamics Understanding of Novel Solvated Drug Molecules Against Emerging *Burkholderia Cepacia* Infections in Immunocompromised Cancer Patients**

Sajjad Ahmad^1*^, Faisal Ahmad^2,3^, Syed Ainul Abideen^4^, Kalsoom Khan^1^, Muhammad Irfan^5^, Farhan Siddique^6^, Norah Abdullah Albekairi^7^, Abdulrahman Mohammed Alshammari^7^, Dong-Qing Wei^8^

^1^Department of Health and Biological Sciences, Abasyn University, Peshawar 25000, Pakistan. [sahmad@bs.qau.edu.pk](mailto:sahmad@bs.qau.edu.pk), sajjad.ahmad@abasyn.edu.pk.

^2^World Health Organization, Park Road, Chak Shahzad, Islamabad, 44000 Pakistan. [faisalahmad@bs.qau.edu.pk](mailto:faisalahmad@bs.qau.edu.pk)

^3^National Institute of Health. Park road, Chak Shahzad. Islamabad, Islamabad 44000, Pakistan.

^4^Department of Biomedical Engineering, Shanghai Jiao Tong University, Shanghai, China. [syedain07@gmail.com](mailto:syedain07@gmail.com).

^5^Department of Oral Biology, University of Florida, USA.

^6^Department of Pharmaceutical Chemistry, Faculty of Pharmacy, Bahauddin Zakriya University, Multan 60800, Pakistan; [drfarhansiddique@bzu.edu.pk](mailto:drfarhansiddique@bzu.edu.pk)

^7^Department of Pharmacology and Toxicology, College of Pharmacy, King Saud University, Post Box 2455, Riyadh, 11451, Saudi Arabia. [Nalbekairi@ksu.edu.sa](mailto:Nalbekairi@ksu.edu.sa), Abdalshammari@ksu.edu.sa

^8^State Key Laboratory of Microbial Metabolism, Joint International Research Laboratory of Metabolic & Developmental Sciences and School of Life Sciences and Biotechnology, Shanghai Jiao Tong University, Shanghai 200030, P.R. China. [dqwei@sjtu.edu.cn](mailto:dqwei@sjtu.edu.cn)

***Correspondence:** [sahmad@bs.qau.edu.pk](mailto:sahmad@bs.qau.edu.pk), [sajjad.ahmad@abasyn.edu.pk](mailto:sajjad.ahmad@abasyn.edu.pk)

***Correspondence:** [sajjad.ahmad@abasyn.edu.pk](mailto:sajjad.ahmad@abasyn.edu.pk)


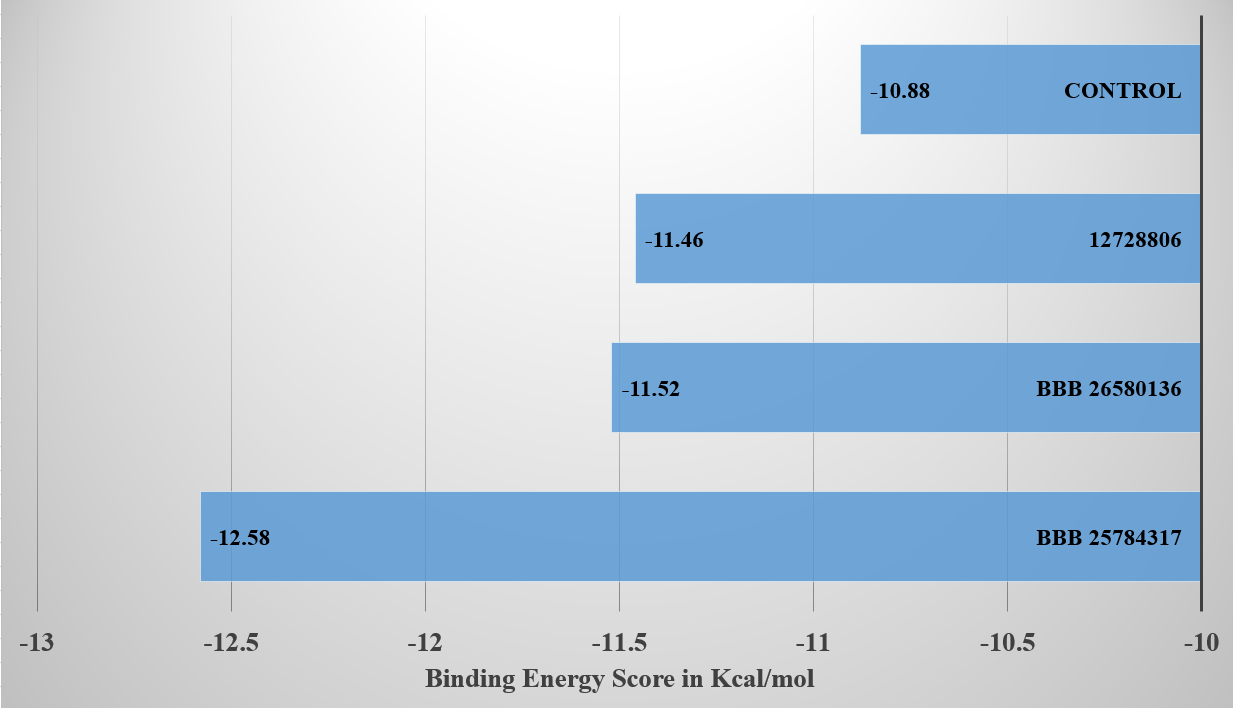


**S-Fig.1.** The predicted binding energy score of compounds for the receptor enzyme. The unit of energy is given in kcal/mol.


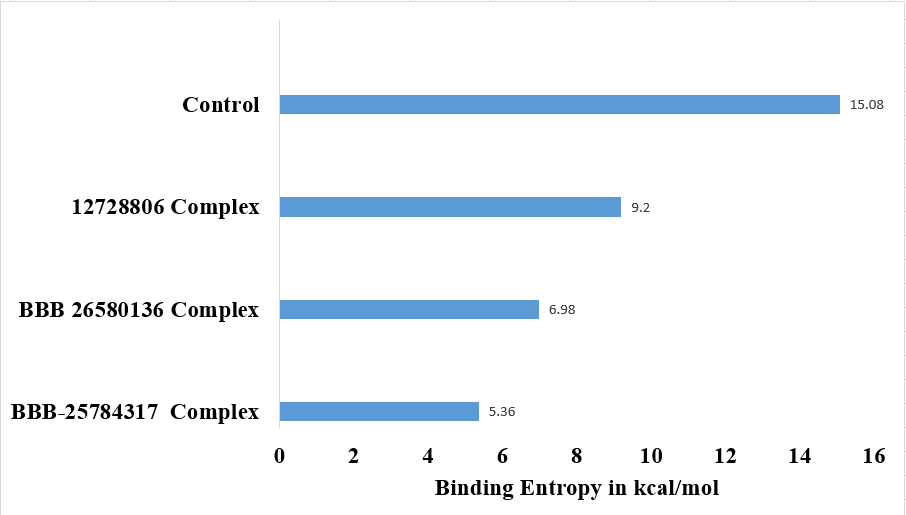


**S-Fig.2.** Binding entropy energy of complexes estimated by AMBER normal mode analysis.


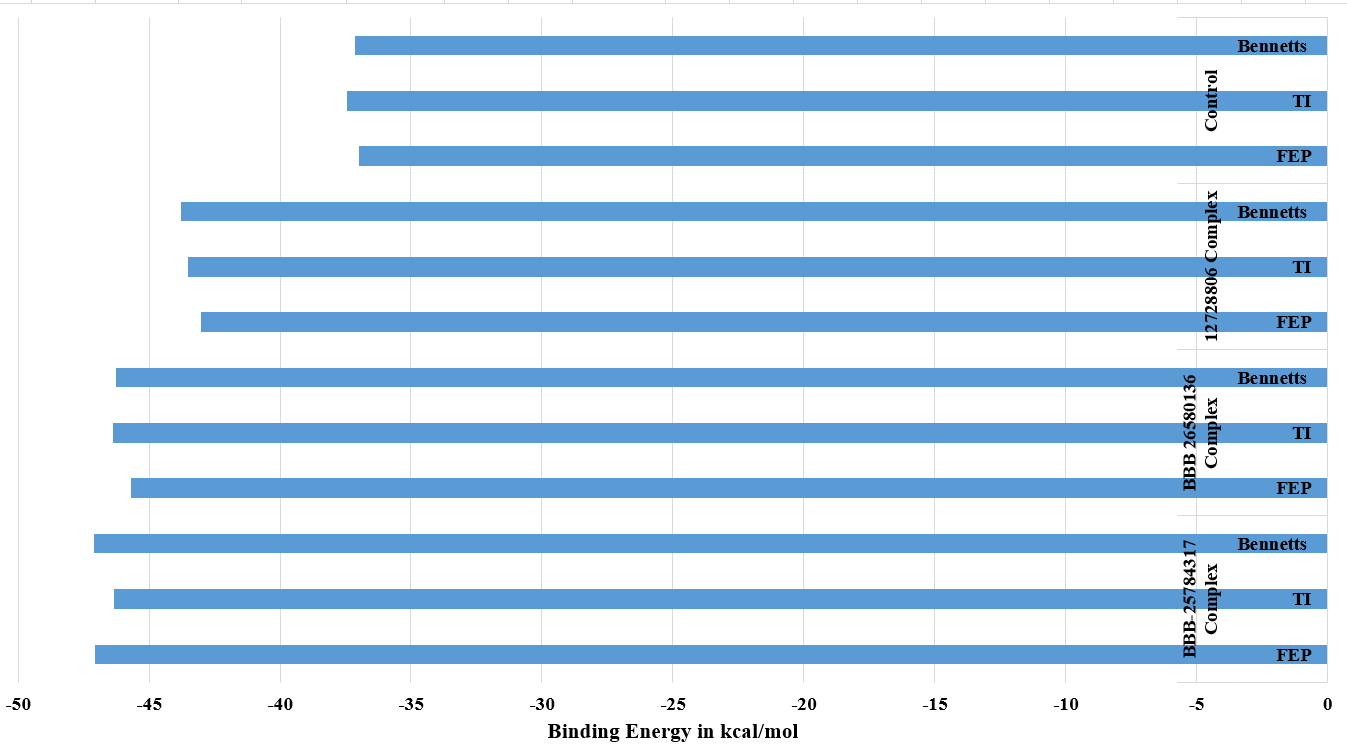


**S-Fig.3.** WaterSwap binding energy of complexes in kcal/mol. Three algorithms were employed including FEP, TI, and Bennett’s.
